# An unusual etiology of posttraumatic Collet–Sicard Syndrome: a case report

**DOI:** 10.11604/pamj.2016.23.143.9143

**Published:** 2016-03-30

**Authors:** Walid Mnari, Mohamed Kilani, Khaled Harrathi, Mezri Maatouk, Jamel Koubaa, Mondher Golli

**Affiliations:** 1 Imaging Department, Fattouma Bourguiba University Hospital, Medical university, Monastir, Tunisia; 2 Neurosurgery Department, Fattouma Bourguiba University Hospital, Medical university, Monastir, Tunisia; 3 Otorhinolaryngology Department, Fattouma Bourguiba University Hospital, Medical University, Monastir, Tunisia

**Keywords:** Cranial nerve palsy, styloid process, fracture, CT scan

## Abstract

Posttraumatic Unilateral paralysis of the last four cranial nerves (IX-XI), known as collet-Sicard syndrome, is rare following closed head injury. A 21-year-old man presented with slurred speech, hoarseness voice and difficulty swallowing his saliva following closed head trauma. The cranial nerve examination revealed left sided severe dysfunction of cranial nerves VII, IX, X, XI, and XII. A CT-Scan of the neck was performed demonstrating a fracture of the left styloid process at the base of the skull. The Magnetic Resonance Imaging showed unusually well seen lower cranial nerves due to nerve edema. The patient was managed conservatively with steroids and regular sessions of neuromuscular and orthophonic rehabilitation. The nutrition had to be administered by gastrostomy since he was unable to swallow. Six months after the injury a total neurological recovery was noted. We present the exceptional case of Collet-Sicard Syndrome caused by styloid process fracture.

## Introduction

Collet-Sicard syndrome (CSS) is a condition showing unilateral paralysis of lower cranial nerves (CN) IX, X, XI, XII [1]. It was first described by Collet, in 1915, in a World War I soldier with a bullet injury in the mastoid region [2]. Sicard, in 1917, described five cases with similar clinical features resulting from bullet trauma [3]. This rare syndrome has been attributed to neoplasms of the skull base, inflammatory and vascular lesions [4]. CSS is rare following closed head injury. Occipital condyle and Jefferson fractures are well documented causes of post traumatic CSS [5, 6]. CSS appearing secondary to a styloid process (SP) fracture has never been reported before. This report describes the unique case of a traumatic styloid process causing palsy of the lower cranial nerves.

## Patient and observation

**Figure 1:**
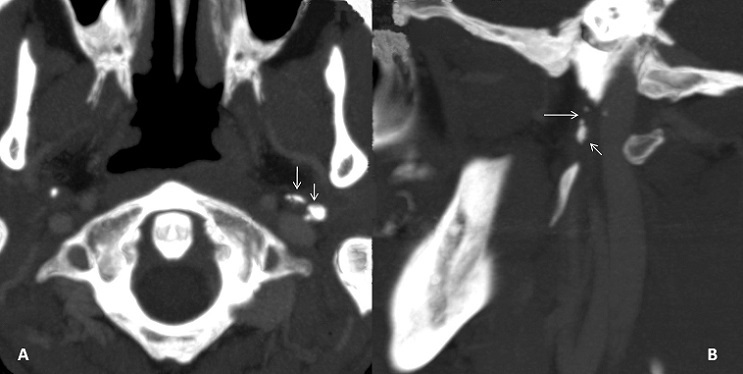
Targeted axial (Panel A) and left sagittal (Panel B) MIP reconstructed CT scan images with bone window of the neck showed a comminute fracture of the styloid process at the base of the skull (arrows)

**Figure 2:**
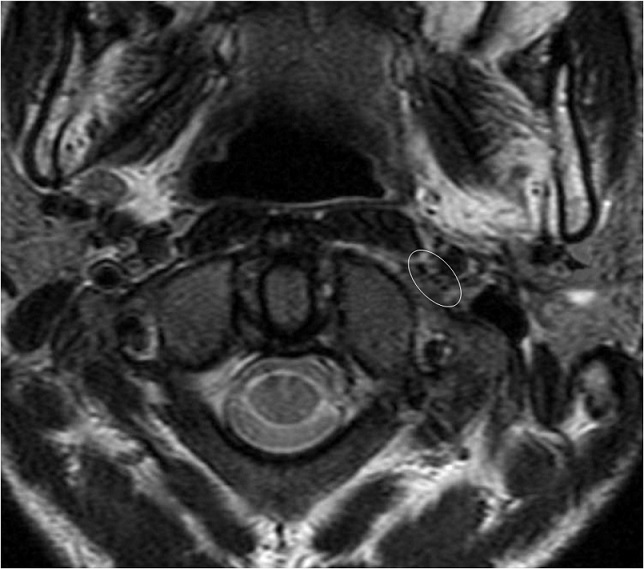
The upper cervical MRI on T2 weighted image demonstrate the fourth lower cranial nerves (circle) which appear as black points with high signal intensity around, related to perinerve oedema. On the other side the CN are not visible

**Figure 3:**
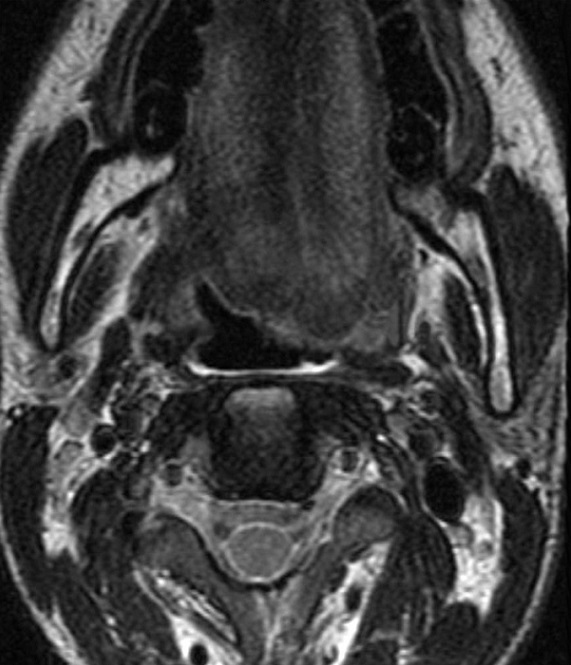
MRI on T2 weighted image showing the drooping and flabby aspects of the left oropharyngeal wall and the left deviation of the tongue

A 21-year-old man presented to our hospital with a trauma of the left cranio-cervical region sustained in a brawl. He suffered of slurred speech, hoarseness voice and difficulty swallowing his saliva. He also complained of left neck pain exacerbated by movement. The physical examination revealed, a left-sided large cervical swelling and a painful limited range of neck motion. No mass was felt in the neck. No carotid bruits were heard and the carotid pulses were symmetric. The cranial nerve examination revealed left sided severe dysfunction of cranial nerves IX, X, XI, and XII. His exam showed a regurgitation of liquid into the nose, asymmetric elevation of the soft palate pulling to the right side, insensate left oropharyngeal wall, left vocal cord paralysis and tongue deviation to the left side with protrusion. The examination of the spinal accessory nerve was limited because of neck pain but there was mild weakness of the left trapezius muscle. The remainder of the neurological examination was normal. The laboratory testing showed no abnormalities. A CT-Scan of the neck was performed demonstrating a fracture of the left styloid process (SP) at the base of the skull (Figure 1). There was a post traumatic high fat density in the deep facial spaces. The adjacent jugulo-carotide vascular axis was permeable. A magnetic resonance imaging (MRI) of the head and neck founded an increased signal intensity and thickening soft tissues surrounding the SP. The fourth lower cranial nerves were unusually well seen and discarded by high signal intensity around (Figure 2). The high signal intensity around uncut nerves was related to nerve edema. The MRI also revealed drooping and flabby aspects of the left oropharyngeal wall and left deviated tongue related to the paralysis of the IX and the XII cranial nerves (Figure 3). The MRI of the brain was normal. The patient was managed conservatively with steroids (300mg of prednisone daily) and analgesics. A nasogastric tube was placed. The patient received regular sessions of neuromuscular and orthophonic rehabilitation. Four weeks later the slurred speech, hoarseness voice and impaired neck were considerably improved. However, he was still unable to swallow and the nutrition had to be administered by gastrostomy. Six months after the injury a total neurological recovery was noted.

## Discussion

Mixed nerves (IX, X, XI) originate from the collateral groove of the posterior bulb. They exit the skull closely associated with one another via the Jugular foramen. The hypoglossal nerve has a vertical origin in the medulla and leaves the midbrain as 10 to 15 sheaths to form a nerve root. It leaves the cranial vault from the hypoglossal canal close to the occipital condyle [6]. Lesions involving mixed nerves at the skull base result in a clinical picture called “Vernet syndrome” or “Jugular Foramen syndrome”. The combination of Vernet syndrome with the twelfth nerve palsy results in “Condylojugular syndrome”, also called “Collet-Sicard Syndrome” [1]. Near their point of exit, CN IX-XII lie between the transverse process of the atlas medially and the SP of the skulllaterally [5]. Zielinski and al, via cadaveric dissection found that the SP was only 8-10 mm from the first cervical vertebra [7]. Multiple CN palsies are often a diagnostic challenge because the nerves can be affected at any site along their course SP fractures appear to be rare [8]. It results generally from macro trauma such as accident, surgery, fight or fall [8]. It may also result from minimal trauma or normal action such as laughing or coughing [9]. These fractures present with symptoms similar to those of “Eagles syndrome” consisting of recurrent throat pain, pharyngeal foreign body sensation, dysphagia, referred ipsilateral otalgia and neck pain [10]. To the best of our knowledge, the onset of CSS following SP fracture has never been reported before. The essential question raised by the present case deals with the hypothesis explaining the lower nerves palsies. The narrow space between the transverse of the atlas and the SP make the lower CN vulnerable in the event of trauma. If the SP is abnormally displaced medially following trauma, this space will be reduced and thereby lower CN palsies will be likely to develop. Hsu and al [4] reported the same ethiopathogenic mechanism of onset of CSS secondary to a displaced atlas fracture. Palsies of the lower CN may result from nerve compression, nerve rootlet avulsion or nerve stretching [4]. Vertebral artery insufficiency and traction injuries have been suggested to explain these CN palsies [11, 12]. The CN injury in our case appeared to be caused by neither brain ischemia, nor direct section by a bony fragment. We presume that the CN were injured by impingement due to soft tissue edema and direct compression by the fractured and displaced SP. The clinical presentation of posttraumatic CSS is highly variable. This syndrome is characterized by hoarseness of the voice, difficulty swallowing, and unclear speech [1, 4, 5]. It is worth mentioning that early detection of this syndrome may be difficult especially in patients with altered state of consciousness [6]. A CT scan with the window level set for bone visualisation, three dimensional CT and volume rendered CT scan are the most useful methods for demonstrating basal fractures of the skull [6, 13]. The extracranial segments of CN IX-XII were consistently demonstrated with MRI [14]. This was further facilitated in this report by the contrast due to the difference between the local edema and the signal void of the adjacent internal carotid artery. The management of CSS involves various strategies which include conservative treatment, medical management or surgical treatment. Surgical intervention may have some risks to the patient [15]. In our case, medical treatment seemed to be a reasonable option. In most cases of posttraumatic CSS, neurological recovery is slow, symptoms improve partially and residual neurologic deficits may persist for a long time [16]. It is also reported that immediate deficits have a lower rate of recovery than secondary deficits [6]. Legros and al, in their review of the literature of reported a rate of 23% of total recovery in patients with CSS secondary to cranial base fractures. In our case, total neurological recovery was noted confirming the published results.

## Conclusion

This is the unique case of Collet Sicard syndrome following an isolated styloid process fracture. It is very uncommon case since it demonstrates that the lower cranial nerves can be damaged by a medially displaced styloid process fracture. This report highlighted the anatomical relation of skull base, upper cervical spine and lower cranial nerves. The investigation of choice is computed tomography of the skull base where the fracture can be sought using serial coronal and sagittal sections. Although posttraumatic CSS has usually a poor prognosis, our case demonstrates that total recovery is possible.
